# Noninvasive mapping of the redox status of dimethylnitrosamine-induced hepatic fibrosis using *in vivo* dynamic nuclear polarization-magnetic resonance imaging

**DOI:** 10.1038/srep32604

**Published:** 2016-09-02

**Authors:** Takahito Kawano, Masaharu Murata, Fuminori Hyodo, Hinako Eto, Nuttavut Kosem, Ryosuke Nakata, Nobuhito Hamano, Jing Shu Piao, Sayoko Narahara, Tomohiko Akahoshi, Makoto Hashizume

**Affiliations:** 1 Innovation Center for Medical Redox Navigation, Kyushu University, 3-1-1 Maidashi, Higashi-ku,, Fukuoka 812-8582, Japan; 2 Department of Advanced Medical Initiatives, Faculty of Medical Sciences, Kyushu University, 3-1-1 Maidashi, Higashi-ku,, Fukuoka 812-8582, Japan.; 3 Center for Advanced Medical Innovation, Kyushu University, 3-1-1 Maidashi, Higashi-ku, Fukuoka 812-8582, Japan; 4 Faculty of Pharmaceutical Science, University of British Columbia, Vancouver, British Columbia, V6T 1Z3, Canada

## Abstract

Hepatic fibrosis is a chronic disorder caused by viral infection and/or metabolic, genetic and cholestatic disorders. A noninvasive procedure that enables the detection of liver fibrosis based on redox status would be useful for disease identification and monitoring, and the development of treatments. However, an appropriate technique has not been reported. This study describes a novel method for assessing the redox status of the liver using *in vivo* dynamic nuclear polarization-magnetic resonance imaging (DNP-MRI) with the nitroxyl radical carbamoyl-PROXYL as a molecular imaging probe, which was tested in dimethylnitrosamine-treated mice as a model of liver fibrosis. Based on the pharmacokinetics of carbamoyl-PROXYL in control livers, reduction rate mapping was performed in fibrotic livers. Reduction rate maps demonstrated a clear difference between the redox status of control and fibrotic livers according to the expression of antioxidants. These findings indicate that *in vivo* DNP-MRI with a nitroxyl radical probe enables noninvasive detection of changes in liver redox status.

Hepatic fibrosis and cirrhosis are caused by the wound-healing response to chronic liver injury, which results from viral hepatitis as well as metabolic and alcoholic liver diseases^1^,^2^,^3^. During fibrogenesis, sustained production of growth factors, cytokines, proteolytic enzymes and angiogenic factors stimulates extracellular matrix deposition, a process that destroys the tissue structure^4^,^5^,^6^,^7^. The prognosis and management of chronic liver disease often depends on the stage of liver fibrosis. Untreated fibrosis may progress to irreversible fibrosis, and induce molecular events that ultimately lead to organ failure and death. Thus, routine assessment and imaging strategies are urgently needed.

To evaluate the severity of hepatic fibrosis and cirrhosis, a liver biopsy may be necessary to establish the diagnosis by histologic inflammatory grade and fibrosis stage^8^,^9^,^10^,^11^. However, drawbacks of biopsies include the risk of complications, intra- and inter-observer variability, inaccurate staging due to sampling error, and the heterogeneous distribution of fibrosis in the liver parenchyma, all of which are not reflected by a single biopsy. Furthermore, the rapid progression of fibrosis, which tends to be non-linear over time, can not be monitored with a single biopsy, and serial biopsies are not an attractive solution for several reasons. To address these issues, noninvasive approaches have been developed such as serum biomarkers of liver fibrosis and ultrasound elastography to measure liver stiffness^12^,^13^,^14^,^15^. Although these approaches are helpful for detecting and diagnosing liver fibrosis, and to frequently monitor disease progression and therapeutic responses, they do not enable the earlier stages of fibrosis to be identified.

The redox status, which is the balance between oxidant and antioxidant agents is a key pathophysiologic factor in numerous liver disorders^16^,^17^. Reactive oxygen species (ROS) play a critical role in the initiation of fibrosis by stimulating the release of profibrogenic growth factors, and cytokines^18^,^19^. This occurs independently of TGF-ß, a redox-sensitive gene, and free radicals lead to increased TGF-ß expression in hepatic stellate cells, which activates collagen-producing cells. Experimental studies indicate that TGF-ß stimulates ROS production in fibroblasts. Furthermore, patients with oxidative stress caused by fibrosis have low levels of antioxidants (e.g., glutathione, superoxide dismutase and catalase)^20^. Therefore, redox status is emerging as an important marker of liver function. Currently available methods do not facilitate noninvasive evaluation of liver functional responses *in vivo*; therefore, an imaging technique for monitoring liver fibrosis based on the local redox environment would be highly valuable for the management of liver disorders and the development of novel therapeutic agents.

*In vivo* dynamic nuclear polarization magnetic resonance imaging (DNP-MRI), also known as proton-electron double resonance imaging or Overhauser MRI, is a technique that allows imaging of the distribution of free radical species within anatomical structures of living animals^21^,^22^,^23^. DNP enhances the MRI signal from nuclei such as ^1^H by irradiating the tissue at the electron paramagnetic resonance (EPR) frequency of the free radical prior to the MRI pulse sequence, thus enhancing the image intensity of free radicals. The redox reaction is modulated by nitroxyl radicals, and the reduction rate depends on the redox environment, which is influenced by factors such as over-production of ROS and reduced antioxidant levels. In a previous study using *in vivo* DNP-MRI with nitroxyl radicals, unique information was obtained on the redox status in living mice^24^,^25^,^26^. Because the results of many *in vitro* studies suggest a relationship between redox balance and liver disorders, a method for noninvasive monitoring of liver redox status, such as DNP-MRI, may represent a powerful tool for understanding the dynamics of redox alterations and for assessing the efficacy of drugs for the treatment of certain liver disorders. Therefore, the objective of this study was to evaluate the redox status in liver fibrosis using *in vivo* DNP-MRI with nitroxyl radicals as molecular imaging probes, and to apply this method to a dimethylnitrosamine (DMN)-treated animal model.

## Results

### Redox imaging using *in vivo* DNP-MRI

**Figure 1:**
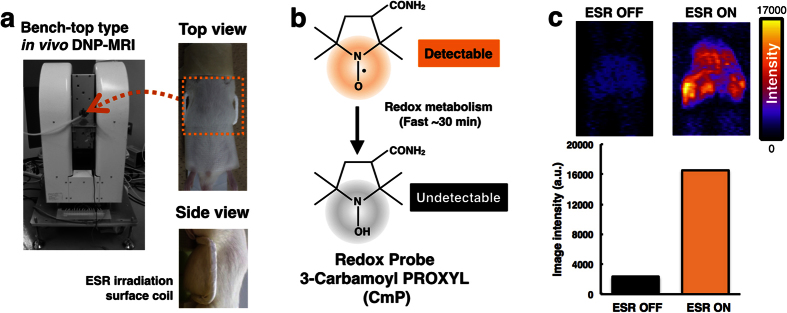
Redox imaging of mouse liver after intravenous injection of nitroxyl radical. (**a**) The experimental set-up using a *in vivo* DNP-MRI (Keller). (**b**) The chemical structure of the redox probe, 3-carbamoyl-PROXYL (carbamoyl-PROXYL), and its nitroxyl reduction by tissue redox status. (**c**) *In vivo* DNP-MRI images of the DMN-treated liver after 1 min injection of carbamoyl-PROXYL, and the plot of image intensity with ESR OFF and ON.

To demonstrate the feasibility of *in vivo* DNP-MRI for liver fibrosis, surface coils were constructed for highly sensitive local imaging (Fig. 1a). The custom-curved surface coils were used to cover the abdomen of mice in all experiments^27^,^28^. Carbamoyl-PROXYL was chosen as the nitroxide imaging probe for determining the redox status in the liver. The chemical structure of carbamoyl-PROXYL is shown in Fig. 1b. Stable nitroxide free radicals and their one-electron reduced products, the hydroxylamines, are recycling antioxidants^29^,^30^. By undergoing one-electron transfer reactions, nitroxyl radicals are reduced to the corresponding hydroxylamine or oxidized to the corresponding oxoammonium cation species. Therefore, nitroxyl radicals are redox-active species, which can be oxidized or reduced by specific reactants in cells and tissues. Nitroxyl radicals are reduced to hydroxylamines by reducing agents such as ascorbate and semiquinone radicals, as well as by intercepting reducing equivalents from the electron transport chain. In this study, carbamoyl-PROXYL, which is thought to be cell permeable, was used in all *in vivo* DNP-MRI experiments. *In vivo* DNP-MRI imaging of mice was performed after intravenous injection of carbamoyl-PROXYL. The signal intensity was clearly enhanced by EPR irradiation (ESR ON) without heating (Fig. 1c).

### Physiologic characteristics and liver pathology

**Figure 2:**
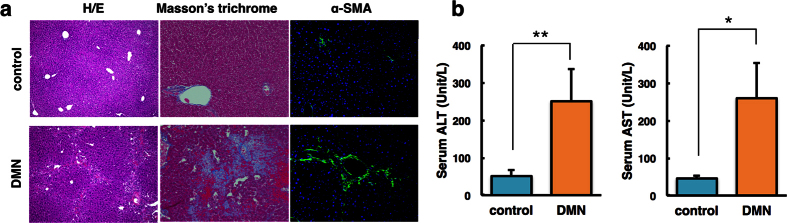
Histologic analysis and assessment of mouse liver tissue after dimethylnitrosamine (DMN) treatment. (**a**) Haematoxylin and eosin (H/E) staining, Masson’s trichrome staining and immunofluorescence staining of α-smooth muscle actin (SMA). Original magnification: 100×, 200× and 200×, respectively. (**b**) Serum levels of alanine transaminase (ALT) and aspartate transaminase (AST) after 4 weeks of DMN treatment versus control. *P < 0.05, **P < 0.01.

Histologic changes in the liver tissue of mice were examined to evaluate the extent of hepatic fibrosis caused by DMN. Intact lobular architecture with central veins and radiating hepatic cords were present in control mice, whereas DMN-treated mice exhibited severe hepatic damage, as indicated by collagen synthesis, sinusoidal congestion, massive necrosis of hepatocytes and inflammation (Fig. 2a). Immunostaining of α-smooth muscle actin (α-SMA), a marker of fibrogenesis, revealed a prominent signal in DMN-treated mice, but a relatively weak signal in control mice.

Mice treated with DMN had a lower body weight than control mice, and had a slightly lower liver weight (Table S1). To assess the effect of DMN on liver function, serum levels of aminotransferases were analysed. As depicted in Fig. 2b, DMN induced a significant increase in alanine transaminase (ALT) and aspartate transaminase (AST) compared with the control (Fig. 2b). The clearance/retention volume of liver function was evaluated by indocyanine green (ICG) in blood at 15 min after injection. DMN-treated mice displayed a significant signal (Fig. S1).

### *In vivo* DNP-MRI measurement of liver fibrosis

**Figure 3:**
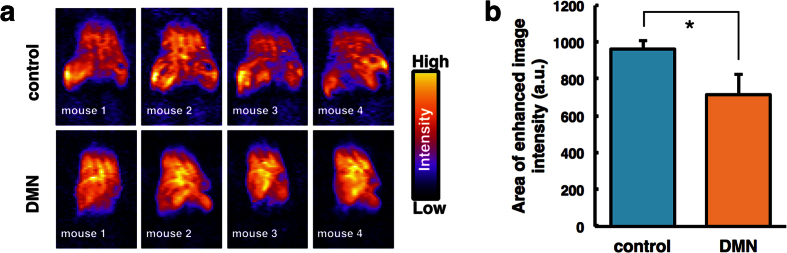
Distribution of carbamoyl-PROXYL in dimethylnitrosamine (DMN)-treated mouse liver after intravenous injection. (**a**) DNP-MRI images of the spatial distribution of carbamoyl-PROXYL as a function of liver area after 1-min injection. (**b**) The plot of the area of enhanced image intensities. *P < 0.01.

Figure 3 shows the radical distribution at 1 min after injection of carbamoyl-PROXYL. The area of enhanced image intensity was reduced in the liver of DMN-treated mice compared with control mice because the liver was became atrophied in DMN-treated mice (see Table S1). *In vivo* DNP-MRI imaging enabled the liver morphology to be visualized.

**Figure 4:**
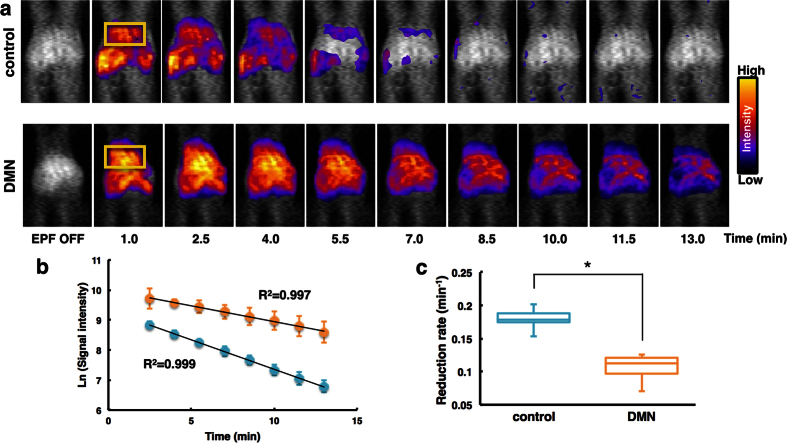
Redox imaging of liver in dimethylnitrosamine (DMN)-treated mice using *in vivo* DNP-MRI. (**a**) Temporal changes in DNP-MRI images of DMN-treated mouse liver after intravenous injection of carbamoyl-PROXYL. ROIs are indicated by the yellow box. (**b**) Time course of the image intensity of nitroxyl radicals of carbamoyl-PROXYL in control mice (blue circles) and DMN-treated mice (orange circles). (**c**) Rate constants of carbamoyl-PROXYL reduction in the liver. *P < 0.01.

To noninvasively monitor the redox status of the liver, *in vivo* DNP-MRI was performed after DMN treatment in living mice (Fig. 4a). At 1 min after injection of carbamoyl-PROXYL, the image intensity was clearly enhanced in the livers of both control and DMN-treated mice. By 10 min after injection, there was a substantial decrease in control mice, whereas only a slight decrease occurred in DMN-treated mice. Figure 4b shows a plot of the time course of the signal intensity of carbamoyl-PROXYL in the liver. The decline in carbamoyl-PROXYL concentrations was linear in both control and DMN-treated mice. Solid lines through the data points indicate a linear fit to the respective data set. The reduction rate in DMN-treated mice (0.11 ± 0.02 min^−1^) was significantly slower than that in control mice (0.18 ± 0.02 min^−1^), as depicted in Fig. 4c.

**Figure 5:**
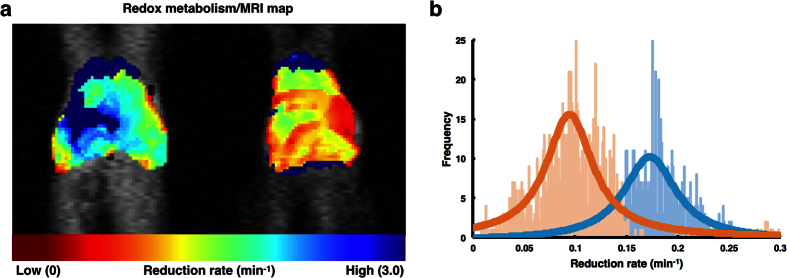
Mapping of redox status of liver in dimethylnitrosamine (DMN)-treated mice. (**a**) Carbamoyl-PROXYL reduction rate mapping in control and DMN-treated mice. The reduction rate of carbamoyl-PROXYL was evaluated per pixel and mapped on MRI images. (**b**) Frequency plot of reduction rates of carbamoyl-PROXYL.

The reduction constant of carbamoyl-PROXYL is likely to depend on the location within the mouse liver. Thus, the reduction rate of carbamoyl-PROXYL per pixel was estimated using the experimental EPR signal intensity data, and reduction rate maps of carbamoyl-PROXYL were calculated. The redox maps clearly demonstrated the site-specific distribution of the reduction rate of carbamoyl-PROXYL in the liver (Figs 5a and S2). In DMN-treated mice, the reduction rate of carbamoyl-PROXYL was significantly slower than that in the control mice (Fig. 5b).

**Figure 6:**
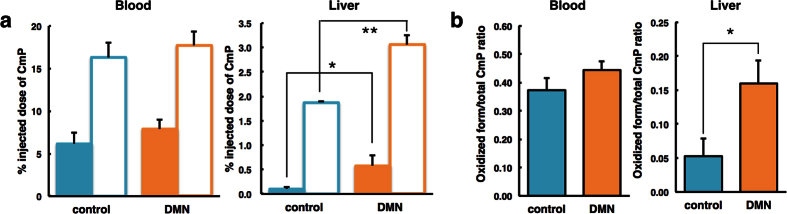
Analysis of the oxidized and reduced form of carbamoyl-PROXYL in mouse blood and liver. (**a**) The distributions of the oxidized form (opaque bar) and total carbamoyl-PROXYL (transparent bar) after a 5-min intravenous injection. Total carbamoyl-PROXYL was measured after the addition of potassium ferricyanide. (**b**) The oxidized/total carbamoyl-PROXYL ratio was determined. Data represent the mean ± SE from three mice per group. *P < 0.05, **P < 0.01.

To confirm that the images directly corresponded to the liver, mice were sacrificed at 5 min after intravenous injection of the probes. The abdominal organs were removed and the blood samples were collected. The samples were homogenized and analysed using conventional X-band EPR spectroscopy (Fig. 6). This *ex vivo* study revealed the no observation the difference of the oxidized and reduced form of carbamoyl-PROXYL in the blood between control and DMN-treated mice. DMN-treated mouse livers had a higher level of residual carbamoyl-PROXYL than those of control livers. DMN-treated mice had decreased clearance. An increase in the oxidized form/total carbamoyl-PROXYL ratio was found in the DMN-treated mouse livers. These results suggested that the slower reduction rates of the carbamoyl-PROXYL in DMN-treated mice were mainly due to the redox reaction in the fibrotic livers.

### Analysis of liver reduction activities

**Figure 7:**
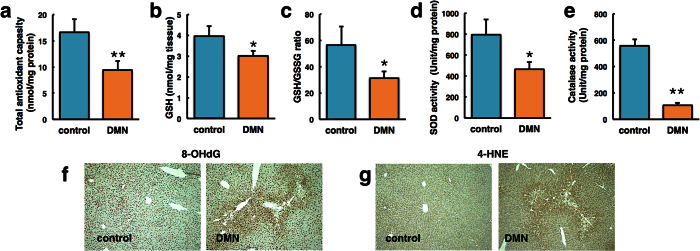
Analysis of liver reductive activity in dimethylnitrosamine (DMN)-treated mice. (**a**) Total antioxidant capacity, (**b**) GSH level, (**c**) GSH/GSSG ratio, (**d**) SOD activity, and (**e**) catalase activity were measured in control and DMN-treated mice. Data represent the mean ± SE from three mice per group. *P < 0.05, **P < 0.01 vs control mice. (**f**) 8-OHdG and (**g**) 4-HNE levels were measured by immunocytochemistry. Original magnification: 100×.

Redox data demonstrated that DMN treatment significantly decreased the rate constant of nitroxide reduction in the liver. Because DMN exposure catalyses an excessive amount of ROS, the antioxidant capacity and molecular markers, including glutathione, superoxide dismutase (SOD) and catalase, in the liver were investigated (Fig. 7a–e). Decreased hepatic antioxidant levels were observed in the DMN-treated mice compared with control mice. DNA and protein carbonylation, which are considered to be oxidative markers, were significantly increased in DMN-exposed compared with control liver tissue (Fig. 7f,g).

## Discussion

Excessive ROS production and reduced antioxidant defences may contribute to numerous degenerative liver diseases. Despite the importance of noninvasive assessment of oxidative stress, the redox status of liver diseases has not been investigated, even *in vitro*. In contrast to these classic methods, the *in vivo* EPR/nitroxide spin-probe method has been used since the early 1990s and has been recognized to be suitable for the examination of free radical reactions in *in vivo* experimental disease models^31^,^32^. When using this approach, selection of the appropriate nitroxide molecule is important because the probe detects the specific locations of these molecules. *In vivo* molecular-level redox imaging was considered suitable to determine the redox status of target organs and to directly assess the activity of pharmaceutical drugs. Using nitroxyl radicals as redox probes, any shift in reduction rate caused by either radical overproduction or defective scavenging systems will be visible as a change in the redox status of living animals.

Changes in signal intensity due to magnetic nitroxide-diamagnetic hydroxylamine conversion is known to reflect the redox state of cells and tissues. Mapping of the reduction rate or half-lives of nitroxide probes on DNP-MRI images can provide useful information on oxidative stress by comparing the reduction capability of nitroxide probes in control livers, as shown in this study. The reduction rate of carbamoyl-PROXYL was visualized in 2D slices, and the distribution of reduction rate was remarkably heterogeneous throughout the tissue area. Previous *in vitro* evidence indicates that the reduction rate of redox probes is strongly affected by cellular metabolism due to their stability in blood, suggesting that the behaviour of redox probes is determined predominantly in the tissue compartment^33^. Therefore, more sophisticated imaging techniques are required to directly determine the pharmacokinetic behaviour of probes in specific organs.

Nitroxide levels were lower in the fibrotic mouse liver than in control mouse livers. This finding may be explained by an imbalance in redox status due to liver fibrosis (Figs 4 and 5). The liver has a specialized defence mechanism to scavenge ROS, in which nuclear factor E2-related factor 2 (Nrf-2) plays an central role^34^,^35^,^36^. Nrf-2 acts as a cellular sensor of redox status and promotes antioxidant system activation^37^. DMN treatment is thought to induce liver damage by causing oxidative stress, which is established to be a critical contributor to the development of hepatic fibrogenesis. It has been shown that DMN has the capacity to damage Nrf-2, which inactivates the Nrf2/ARE pathway and stimulates the expression of multiple antioxidant enzymes^38^. As a result, DMN augments oxidative damage by decreasing levels of SOD and catalase. Studies in different animal models have indicated that the Nrf-2/ARE pathway counteracts viral hepatitis, nonalcoholic fatty liver disease (NAFLD), nonalcoholic steatohepatitis (NASH) and cancer by activating gene expression^39^. Moreover, this pathway supports liver regeneration. Nrf-2 knockout increases liver damage in response to toxins and a high-fat diet, consequently leading to elevated mitochondrial ROS production. Mitochondrial biogenesis is an important determinant of tissue redox status due to electron transfer for ATP and free radical production^40^,^41^. It is thought that increased mitochondrial biogenesis in DMN-treated animal models may partially underlie changes in redox status. Research has expanded knowledge of the mechanisms of oxidative stress, and DNP-MRI may enable the design of new therapies for other liver disorders connected to redox state such as NAFLD and NASH, which are difficult to assess by serum biomarkers.

*Ex vivo* analysis showed that clearance of carbamoyl-PROXYL was lower in fibrotic livers. This is in concordance with the ICG test, which indicated that liver metabolism was impaired in DMN-treated mice. The *ex vivo* experiments also suggested that the reducing activity of the DMN-treated liver was diminished, consistent with *in vivo* data on the oxidized form/total carbamoyl-PROXYL ratio. Because carbamoyl-PROXYL is water soluble and excreted via the urine, changes in renal plasma flow or the glomerular filtration rate may alter its signal decay rate^42^. The redox maps for carbamoyl-PROXYL demonstrated synergistically contributed the clearance/retention and hepatic reducing activity. Clinical evaluation of liver function requires blood tests to assess the status of hepatocytes and the biliary tract, clearance/retention tests such as ICG, and scintigraphy techniques such as galactosyl human serum albumin (GSA) labelled with ^99m^Technetium (^99m^Tc)^43^,^44^. Although clearance/retention tests and scintigraphy have some similarities, the latter technique is less popular. DNP-MRI is a promising approach for comprehensive evaluation of liver function using the combination of *in vivo* redox mapping and *ex vivo* analyses.

A recent study used the DNP-MRI technique, in combination with nitroxyl radicals possessing different structures and physicochemical properties, for spatiotemporal mapping of redox status, pH and oxygen concentration in various animal models^45^,^46^,^47^,^48^. For *in vivo* redox imaging, DNP-MRI was confirmed to be feasible for noninvasive visualization of the distribution and elimination of redox probes in the body. Based on this technique, changes in image intensity as a function of time and location have the potential to reflect the presence of free radicals and oxidative stress, thus providing a quantitative measure of the local redox status of target organs.

In the present study, *in vivo* DNP-MRI using nitroxyl radicals and a customized surface coil resulted in visualization of mouse liver tissue. *In vivo* DNP-MRI enables analysis of the redox environment of fibrotic mouse liver after DMN treatment. The redox maps and decay rates clearly identified local redox changes as well as differences between control and DMN-treated mice. The ability of *in vivo* DNP-MRI to noninvasively detect the focal redox status of the liver suggests that it has the potential to be a valuable tool for the diagnosis and monitoring of liver disorders, and preclinical assessment of novel treatment strategies.

## Materials and Methods

### Animal experiments

Male 5-week-old balb/c mice (KBT Oriental, Ltd., Saga, Japan) were maintained in a 12-h light/dark cycle, and provided with water and food *ad libitum*. Mice were intraperitoneally injected with 10 mg/kg of DMN (N-nitrosodimethylamine; Wako Pure Chemical Ind. Ltd., Osaka, Japan) in 0.9% physiologic saline twice a week for 4 weeks. All animal care and experimental procedures were approved by the committee on the Ethics of Animal Experiments, Kyushu University, and were conducted in accordance with the Guidelines for Animal Experiments of Kyushu University.

### Serum biochemical analysis

At least three mice were sacrificed after 4 weeks of DMN treatments to collect blood. Serum ALT and AST levels were determined with the Fuji DRI-CHEM NX500V system (Fuji Film, Tokyo, Japan) according to the manufacturer’s instructions.

### Histological analysis

After fixing in 10% neutral-buffered formalin, liver tissue was paraffin-embedded, and sliced into 5-μm sections. For histologic assessment, the sections were stained with haematoxylin-eosin and Masson’s trichrome. Immunohistochemistry was performed with anti-α-smooth muscle actin antibodies (Abcam, Cambridge, UK) and the sections were visualized under a fluorescence microscope (BioRevo BZ-9000; Keyence, Osaka, Japan).

### *In vivo* DNP-MRI measurement

Redox imaging was performed with low field type of *in vivo* DNP-MRI system (Keller, Japan Redox Inc. Japan). The external magnetic field B0 for EPR irradiation and MRI was fixed at 16 mT, and the frequencies of EPR irradiation and MRI were 455 MHz and 683 kHz, respectively. For EPR irradiation during liver imaging, a rectangular, one-turn curved surface coil (longitudinal 20 mm, lateral 32 mm) was constructed. In *in vivo* experiments, mice were anaesthetized with 2% isoflurane and secured in sternal recumbency on a specialized holder with adhesive skin tape. The holder was placed in the resonator and *in vivo* DNP-MRI imaging of the upper abdomen was started immediately after intravenous administration of carbamoyl-PROXYL (150 mM, 10 μL/Kg). Pharmacokinetic DNP-MRI images were obtained at several time points from 1 to 13 min after injection. Normal MRI images were obtained without EPR irradiation. The *in vivo* redox map was generated from the slope of the image intensity of each pixel in four pharmacokinetic images using a customized Excel macro program. The scanning conditions for *in vivo* DNP-MRI were as follows: power of EPR irradiation, 7 W; flip angle, 90°; repetition time (TR) × echo time (TE) × EPR irradiation time (TEPR), 500 × 25 × 250 ms; number of averages, 2; slice thickness, 100 mm, including the entire width of each mouse; phase-encoding steps, 32; field of view (FOV), 40 × 40 mm; and matrix size, 64 × 64 after reconstruction.

### Assessment of nitroxyl radicals concentration in liver and blood

A carbamoyl-PROXYL (10 μL/g body weight of 150 mM) solution was intravenously administered to control or DMN-treated mice. A liver and blood sample were collected at 5 min after injection, and the radical and total nitroxyl radical reoxidized by potassium ferricyanide (final 2 mM) concentration in the blood and liver were measured by X-band EPR after liver sample was homogenized by PBS.

### Measurement of reduction activities

Liver tissues were homogenized in lysis buffer, centrifuged, and the supernatants were collected. The total antioxidant capacity (TAC), GSH, SOD and catalase activity in tissue homogenates were analysed using an OxiSelect Total Antioxidant Capacity Assay Kit (Cell Biolabs Inc., San Diego, CA, USA), a GSSG/GSH Quantification Kit (Dojindo Laboratories, Kumamoto, Japan), a SODAssay Kit-WST (DOJINDO) and a catalase colorimetric assay kit (ARBOR ASSAYS, Bangor, ME, USA), respectively, according to the standard protocols. The samples were analysed using the microplate reader EnSpire 2300 Multilabel (PerkinElmer Inc., Waltham, MA, USA).

### Statistical analysis

Differences between the groups of mice were analysed using the unpaired Student’s *t*-test. *P*-values less than 0.05 were considered to denote statistical significance. Results are expressed as the means ± SD.

## Additional Information

**How to cite this article**: Kawano, T. *et al.* Noninvasive mapping of the redox status of dimethylnitrosamine-induced hepatic fibrosis using *in vivo* dynamic nuclear polarization-magnetic resonance imaging. *Sci. Rep.* **6**, 32604; doi: 10.1038/srep32604 (2016).

## Supplementary Material

Supplementary Information

## References

[b1] Sherlock S. & Dooley J. Hepatic cirrhosis, 10^th^ ed. In Disease of the liver and biliary system. Oxford: Backwell Science Ltd. 371–384 (1997).

[b2] Cohen J. C., Horton J. D. & Hobbs H. H. Human fatty liver disease: old questions and new insights. Science 332, 1519–1523 (2011). 21700865 10.1126/science.1204265 PMC3229276

[b3] Poynard T., Bedossa P. & Opolon P. Natural history of liver fibrosis progression in patients with chronic hepatitis C. Lancet 349, 825–832 (1997). 9121257 10.1016/s0140-6736(96)07642-8

[b4] Friedman S. L. The cellular basis of hepatic fibrosis. N. Engl. J. Med. 328, 1828–1835 (1993). 8502273 10.1056/NEJM199306243282508

[b5] Friedman S. L. Molecular regulation of hepatic fibrosis, an integrated cellular response to tissue injury. J. Biol. Chem. 275, 2247–2250 (2000). 10644669 10.1074/jbc.275.4.2247

[b6] Pellicoro A., Ramachandran P., Iredale J. P. & Fallowfield J. A. Liver fibrosis and repair: immune regulation of wound healing in a solid organ. Nat. Rev. Immunol. 14, 181–194 (2014). 24566915 10.1038/nri3623

[b7] Rojkind M. & Martinez-Palomo A. Increase in type I and type III collagens in human alcoholic liver cirrhosis. Proc. Natl. Acad. Sci. USA 73, 539–543 (1976). 1061156 10.1073/pnas.73.2.539 PMC335945

[b8] Rockey D. C., Caldwell S. H., Goodman Z. D., Nelson R. C. & Smith A. D. American association for the study of liver diseases. Liver biopsy. Hepatology 49, 1017–1044 (2009). 19243014 10.1002/hep.22742

[b9] Teixeira-Clerc F. *et al.* CB1 cannabinoid receptor antagonism: a new strategy for the treatment of liver fibrosis. Nat. Med. 12, 671–676 (2006). 16715087 10.1038/nm1421

[b10] Dienstag J. L. The role of liver biopsy in chronic hepatitis C. Hepatology 36, 152–160 (2002). 10.1053/jhep.2002.36381 12407589

[b11] Bedossa P., Dargere D. & Paradise V. Sampling variability of liver fibrosis in chronic hepatitis C. Hepatology 38, 1449–1457 (2003). 14647056 10.1016/j.hep.2003.09.022

[b12] Castéra L. *et al.* Prospective comparison of transient elastography, Fibrotest, APRI, and liver biopsy for the assessment of fibrosis in chronic hepatitis C. Gastroenterology 128, 343–350 (2005). 15685546 10.1053/j.gastro.2004.11.018

[b13] Sebastiani G. & Alberti A. How far is noninvasive assessment of liver fibrosis from replacing liver biopsy in hepatitis C? J.Viral Hepat. 19, 18–32 (2012). 22233410 10.1111/j.1365-2893.2011.01518.x

[b14] Castéra L. *et al.* Pitfalls of liver stiffness measurement: a 5-year prospective study of 13,369 examinations. Hepatology 51, 828–835 (2010). 20063276 10.1002/hep.23425

[b15] Blomley M. J. *et al.* Liver microbubble transit time compared with histology and Child-Pugh score in diffuse liver disease: a cross sectional study. Gut 52, 1188–1193 (2003). 12865280 10.1136/gut.52.8.1188 PMC1773750

[b16] Zhu R., Wang Y., Zhang L. & Guo Q. Oxidative stress and liver disease. Hepatol. Res. 42, 741–749 (2012). 22489668 10.1111/j.1872-034X.2012.00996.x

[b17] Muriel P. Role of free radicals in liver diseases. Hepatol. Int. 3, 526–536 (2009). 19941170 10.1007/s12072-009-9158-6 PMC2790593

[b18] Césaratto L., Vascotto C., Calligaris S. & Tell G. The importance of redox state in liver damage. Ann. Hepatol. 3, 86–92 (2004). 15505592

[b19] Lee J., Giordano S. & Zhang J. Autophagy, mitochondria and oxidative stress: cross-talk and redox signalling. Biochem. J. 441, 523–540 (2012). 22187934 10.1042/BJ20111451 PMC3258656

[b20] Edeas M., Attaf D., Mailfert A. S., Nasu M. & Joubet R. Maillard reaction, mitochondria and oxidative stress: potential role of antioxidants. Pathol. Biol. (Paris) 58, 220–225 (2010). 20031340 10.1016/j.patbio.2009.09.011

[b21] Overhauser A. W. Polarization of Nuclei in Metals. Phys. Rev. 92, 411–415 (1953).

[b22] Lurie D. J., Bussell D. M., Bell L. H. & Mallard J. R. Proton-electron double magnetic resonance imaging of free radical solutions. J. Magn. Reson. 76, 366–370 (1988).

[b23] Eto H. *et al.* Redox imaging of skeletal muscle using *in vivo* DNP-MRI and its application to an animal model of local inflammation. Free Radic. Biol. Med. 89, 1097–1104 (2015). 26505925 10.1016/j.freeradbiomed.2015.10.418

[b24] Ilangovan G. *et al.* *In vivo* measurement of regional oxygenation and imaging of redox status in RIF-1 murine tumor: Effect of carbogen-breathing. Magn. Reson. Med. 48, 723–730 (2002). 12353291 10.1002/mrm.10254

[b25] Kosem N. *et al.* Whole-body kinetic image of a redox probe in mice using Overhauser-enhanced MRI. Free Radic. Biol. Med. 53, 328–336 (2012). 22579576 10.1016/j.freeradbiomed.2012.04.026

[b26] Utsumi H. *et al.* Simultaneous molecular imaging of redox reactions monitored by Overhauser-enhanced MRI with 14N- and 15N-labeled nitroxyl radicals, Proc. Natl. Acad. Sci. USA 103, 1463–1468 (2006). 16432234 10.1073/pnas.0510670103 PMC1345719

[b27] Yamato M. *et al.* Overhauser-enhanced magnetic resonance imaging characterization of mitochondria functional changes in the 6-hydroxydopamine rat model. Neurochem. Int. 59, 804–811 (2011). 21871513 10.1016/j.neuint.2011.08.010

[b28] Matsumoto S. *et al.* Advantageous application of a surface coil to EPR irradiation in overhauser-enhanced MRI. Magn. Reson. Med. 57, 806–811 (2007). 17390363 10.1002/mrm.21198

[b29] Kuppusamy P. *et al.* Noninvasive imaging of tumor redox status and its modification by tissue glutathione levels. Cancer Res. 62, 307–312 (2002). 11782393

[b30] Yamada K. *et al.* Feasibility and assessment of non-invasive *in vivo* redox status using electron paramagnetic resonance imaging. Acta Radiologica 43, 433–440 (2002). 12225490 10.1080/j.1600-0455.2002.430418.x

[b31] Berliner L. J., Fujii H., Wan X. M. & Lukiewicz S. J. Feasibility study of imaging a living murine tumor by electron paramagnetic resonance. Magn. Reson. Med. 4, 380–384 (1987). 3035320 10.1002/mrm.1910040410

[b32] Kuppusamy P. *et al.* Three-dimensional spectral-spatial EPR imaging of free radicals in the heart: a technique for imaging tissue metabolism and oxygenation. Proc. Natl. Acad. Sci. USA 91, 3388–3392 (1994). 8159757 10.1073/pnas.91.8.3388 PMC43582

[b33] Couet W. R. *et al.* Pharmacokinetics and metabolic fate of two nitroxides potentially useful as contrast agents for magnetic resonance imaging. Pharm. Res. 1, 203–209 (1984). 24277329 10.1023/A:1016317212601

[b34] Aleksunes L. M. & Manautou J. E. Emerging role of Nrf2 in protecting against hepatic and gastrointestinal disease. Toxicol. Pathol. 35, 459–473 (2007). 17562481 10.1080/01926230701311344

[b35] Collins A. R. *et al.* Myeloid deletion of nuclear factor erythroid 2-related fac- tor 2 increases atherosclerosis and liver injury. Arterioscler. Thromb. Vasc. Biol. 32, 2839–2846 (2012). 23023374 10.1161/ATVBAHA.112.300345 PMC4490864

[b36] Shin S. M., Yang J. H. & Ki S. H. Role of the Nrf2-ARE pathway in liver diseases. Oxid. Med. Cell. Longev. 2013, 763257 (2013). 23766860 10.1155/2013/763257 PMC3665261

[b37] de Vries H. E. *et al.* Nrf2-induced antioxidant protection: a promising target to counteract ROS-mediated damage in neurodegenerative disease? Free Radic. Biol. Med. 45, 1375–1383 (2008). 18824091 10.1016/j.freeradbiomed.2008.09.001

[b38] Pan T. L. *et al.* Herbal formula, Scutellariae radix and Rhei rhizoma attenuate dimethylnitrosamine-induced liver fibrosis in a rat model. Sci. Rep. 5, 11734 (2015). 26133262 10.1038/srep11734 PMC4488958

[b39] Rolo A. P., Teodoro J. S. & Palmeira C. M. Role of oxidative stress in the pathogenesis of nonalcoholic steatohepatitis. Free Radic. Biol. Med. 52, 59–69 (2012). 22064361 10.1016/j.freeradbiomed.2011.10.003

[b40] Wang S. *et al.* Metabolic factors in the development of hepatic steatosis and altered mitochondrial gene expression *in vivo*. Metabolism 60, 1090–1099 (2011). 21310443 10.1016/j.metabol.2010.12.001

[b41] Murphy M. P. How mitochondria produce reactive oxygen species. Biochem. J. 417, 1–13 (2009). 19061483 10.1042/BJ20081386 PMC2605959

[b42] Ueda A. *et al.* *In vivo* temporal EPR imaging for estimating the kinetics of a nitroxide radical in the renal parenchyma and pelvis in rats. Magn. Reson. Imaging 20, 77–82 (2002). 11973032 10.1016/s0730-725x(02)00467-8

[b43] Morris-Stiff G., Gomez D. & Prasad R. Quantitative assessment of hepatic function and its relevance to the liver surgeon. J. Gastrointest. Surg. 13, 374–385 (2009). 18622661 10.1007/s11605-008-0564-1

[b44] Kwon A.-H., Ha-Kawa S. K., Uetsuji S., Kamiyama Y. & Tanaka Y. Use of technetium 99 m diethylenetriaminepentaacetic acid-galactosyl-human serum albumin liver scintigraphy in the evaluation of preoperative and postoperative hepatic functional reserve for hepatectomy. Surgery 117, 429–434 (1995). 7716725 10.1016/s0039-6060(05)80063-7

[b45] Hyodo F. *et al.* The relationship between tissue oxygenation and redox status using magnetic resonance imaging. Int. J. Oncol. 41, 2103–2108 (2012). 23007796 10.3892/ijo.2012.1638 PMC3583655

[b46] Matsumoto S. *et al.* *In vivo* imaging of tumor physiological, metabolic, and redox changes in response to the anti-angiogenic agent sunitinib: longitudinal assessment to identify transient vascular renormalization. Antioxid, Redox Sign. 21, 1145–1155 (2014). 10.1089/ars.2013.5725 PMC4142786 24597714

[b47] Bobko A. A. *et al.* *In vivo* monitoring of pH, redox status, and glutathione using L-band EPR for assessment of therapeutic effectiveness in solid tumors. Magn. Reson. Med. 67, 1827–1836 (2012). 22113626 10.1002/mrm.23196 PMC3305854

[b48] Koonjoo N. *et al.* *In vivo* Overhauser-enhanced MRI of proteolytic activity. Contrast Media Mol. Imaging 9, 363–371 (2014). 24729587 10.1002/cmmi.1586

