# Supplementary material for: Noninvasive mapping of the redox status of dimethylnitrosamine-induced hepatic fibrosis using in vivo dynamic nuclear polarization-magnetic resonance imaging
Source: Sci Rep. 2016 Sep 2;6:32604. doi: 10.1038/srep32604 (PMC5009327; doi:10.1038/srep32604)
Supplement: Supplementary Information [file srep32604-s1.pdf]

SUPPLEMENTARY INFORMATION

FOR

Noninvasive mapping of the redox status of dimethylnitrosamine-induced hepatic fibrosis using *in vivo* dynamic nuclear polarization-magnetic resonance imaging

Takahito Kawano[1,2], Masaharu Murata[1,2,3,*], Fuminori Hyodo[1,*], Hinako Eto[1], Nuttavut Kosem[1], Ryosuke Nakata[2,3], Nobuhito Hamano[4], Jing Shu Piao[2,3], Sayoko Narahara[1,2], Tomohiko Akahoshi[2] & Makoto Hashizume[1,2,3]

[1]Innovation Center for Medical Redox Navigation, [2]Department of Advanced Medical Initiatives, Faculty of Medical Sciences and [3]Center for Advanced Medical Innovation, Kyushu University, 3-1-1 Maidashi, Higashi-ku, Fukuoka 812-8582, Japan. [4]Faculty of Pharmaceutical Science, University of British Columbia, Vancouver, British Columbia, V6T 1Z3, Canada.

Table S1. Anthropometric parameters of dimethylnitrosamine (DMN)-treated mice

	Control	DMN
Body weight (g)	24.8±2.0	20.8±2.0**
Liver weight (g)	1.14±0.16	0.82±0.12**
Liver/body weight	0.046±0.004	0.037±0.006*

*P<0.05, **P<0.01 compared with control mice.

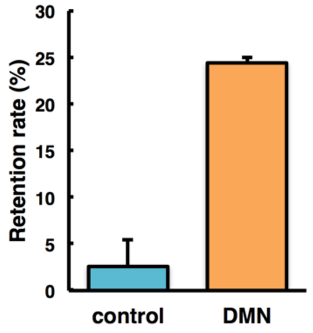

Figure S1. Evaluation of liver clearance/retention function using indocyanine green

The retention volume of indocyanine green in blood was evaluated 15 min after injection.

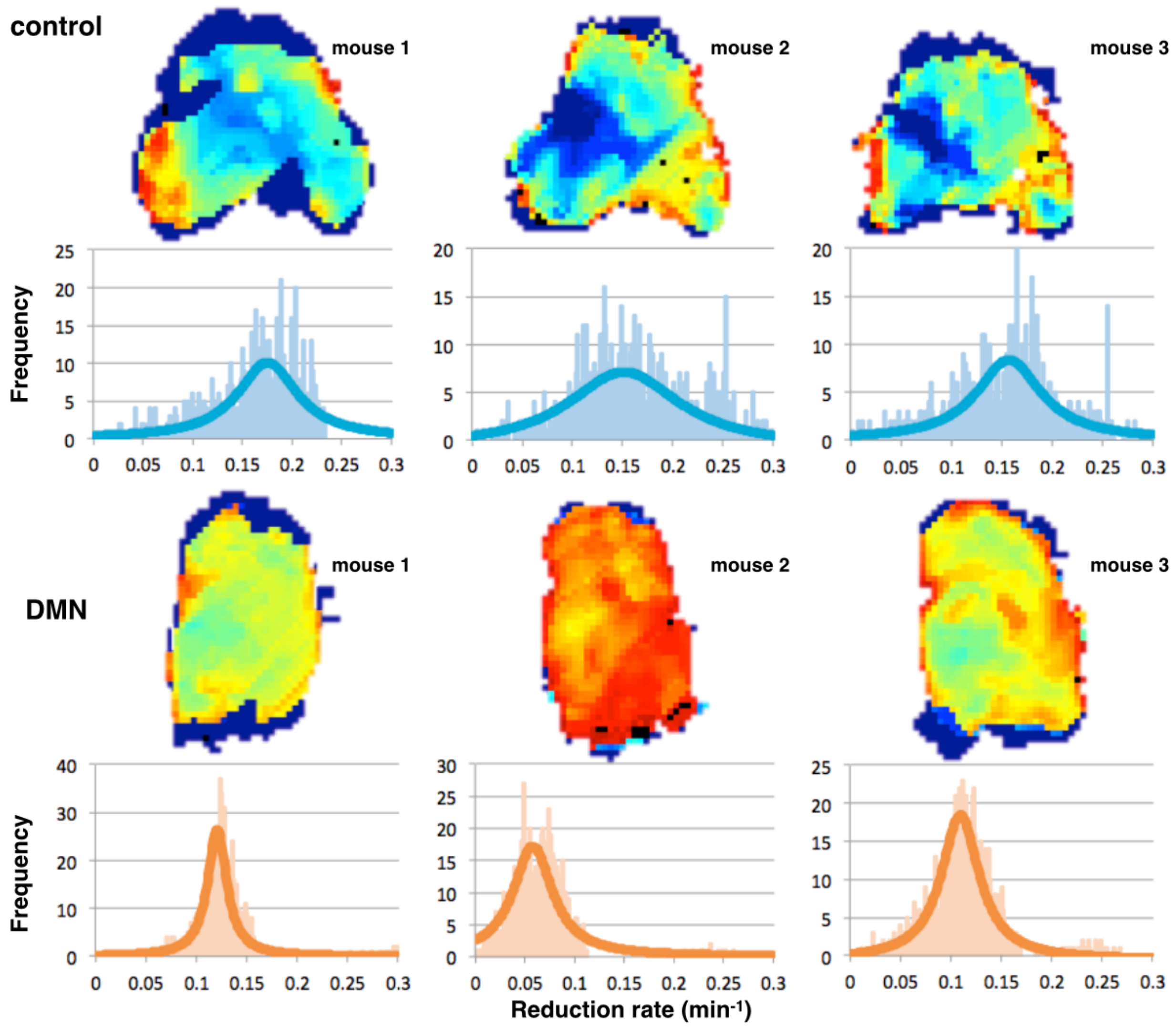

Figure S2. Mapping of liver redox status in dimethylnitrosamine (DMN)-treated mice

Reduction rate mapping of nitroxide carbamoyl-PROXYL in control and DMN-treated mice. The reduction rate of carbamoyl-PROXYL was evaluated per pixel and mapped. Frequency plot of carbamoyl-PROXYL reduction rates.
